# From plate to post: exploring representations of #familymeals through a content analysis of Instagram

**DOI:** 10.1093/heapro/daaf078

**Published:** 2025-06-11

**Authors:** Eloise-kate Litterbach, Emily Denniss, Georgia Middleton

**Affiliations:** Australian Centre for Behavioural Research in Diabetes, Suite G01, 15-31 Pelham Street, Carlton, Victoria 3053, Australia; School of Psychology, Deakin University, 1 Gheringhap St, Geelong, Victoria 3220, Australia; Institute for Health Transformation, Deakin University, 1 Gheringhap St, Geelong, Victoria 3220, Australia; School of Exercise and Nutrition Sciences, Deakin University, Melbourne Burwood Campus, 221 Burwood Highway, Burwood, Victoria 3125, Australia; School of Health and Social Development, Institute for Physical Activity and Nutrition, Deakin University, 1 Gheringhap St, Geelong, Victoria 3220, Australia; College of Nursing and Health Sciences, Flinders University, Caring Futures Institute, Tarntanya, Adelaide, South Australia 5042, Australia

**Keywords:** social media, family meals, nutrition, Instagram, content analysis

## Abstract

Family meals are a popular topic on social media, where people regularly source and share food and nutrition information. However, no research has explored what family meal content is being shared on social media. This study employed a mixed-methods content analysis approach to explore how family meals are portrayed on Instagram. Four hashtags were identified through systematic screening: #familymeals, #familybreakfast, #familylunch, and #familydinner. All post details (video/image, caption, engagement, and account) were collected from the top 15 posts from each hashtag weekly for 14 weeks (February–May 2024). Data were analyzed using a coding framework in REDCap. A total of 564 posts from 359 unique accounts were included. Most account holders were women (86.3%). Recipe developers were the most common account type (38.4%). Most posts depicted food/drink (92.9%), predominantly plated meals (86.6%) and core foods (76.7%), and appeared staged (64.7%). Many captions included meal ideas (70.6%) linking out to or providing recipes (40% and 38.4%) and were described as “quick” or “easy” (38.9%). Differences in post and caption content across hashtags indicated perceptions of what family meals *should* look like depending on time of day, e.g. home-cooked meal at dinner, discretionary food at breakfast, family bonding at lunch. While some information provided in these hashtags may be useful for parents (e.g. quick and easy recipes), the portrayal of perfect meals and mealtimes may perpetuate harmful expectations. Further research is needed to understand how these representations impact parents, and how Instagram can be used to promote realistic, healthy family meals across the day.

Contribution to Health Promotion

- Family meals can be a health-promoting activity, and many parents use social media platforms, such as Instagram, for nutrition information and advice related to mealtimes.
- With no regulation on what is posted, or who is posting on Instagram, we do not know what helpful or harmful family mealtime information or messages parents may be exposed to.
- This paper provides insight into what family meals messages and norms parents are potentially being exposed to on Instagram, and how this may help or hinder efforts for achieving health-promoting family meals.

## INTRODUCTION

Social media has become a huge part of contemporary society, influencing all aspects of life, including what and how we eat. Globally, people spend significant amounts of time on social media, with an average user spending almost two and a half hours per day (We Are Social & Meltwater 2024). Instagram has 1.7 billion users, making it one of the most used social media platforms, with food and nutrition being popular and widely discussed topics on the platform (Muralidhara and Paul 2018, Denniss *et al*. 2023a, We Are Social & Meltwater 2024). Research indicates that individuals actively seek food and nutrition information on Instagram and are also unintentionally and passively exposed to it (e.g. when scrolling their social media feeds, watching Reels, etc.) (Lambert *et al*. 2019, Tricas-Vidal *et al*. 2022, Kreft *et al*. 2023). In a survey of adults from the United States, 44% reported that they follow nutrition influencers on Instagram (Tricas-Vidal *et al*. 2022), in South Africa, 53% of university students follow nutrition pages, and 17% actively seek nutrition information from social media, with Instagram being one of the most popular platforms (Kreft *et al*. 2023). Along with sharing images, videos and descriptive captions, Instagram utilizes hashtags to create key words, which can increase post and account exposure (Gandola 2022). Hashtags related to food and nutrition can cover anything from mealtimes (e.g. #dinnertime) and contexts (e.g. #withfriends), to food descriptions (e.g. #crunchy and #delicious), food groups (e.g. #vegetables), and specific foods (e.g. #peasandcorn). Therefore, the number of potential hashtags related to food and nutrition may be unlimited, and posts with such hashtags may be visible to a vast number of Instagram users.

Family environments and behaviors, which influence children’s dietary intake, health and wellbeing, such as parental feeding practices, food availability and family mealtimes are no longer simply influenced by historical factors such as intergeneration and peers (Mertens *et al*. 2024), but by broader societal factors, including social media (Frey *et al*. 2022). Potential influences of social media can be explained through social cognitive theory, which posits that the external environment (e.g. social media and social norms), personal factors, and behavior are linked and influence each other, and people learn through observation, which can occur via social media (Bandura 1986). Parents and caregivers regularly share or search for information, ideas, inspiration and advice about feeding children (Dworkin *et al*. 2013, Moon *et al*. 2019, Frey *et al*. 2022), and social media is a popular channel for parents and caregivers to both source and share health information for their families (Duggan *et al*. 2015, Pretorius *et al*. 2019, Kubb and Foran 2020, Virani *et al*. 2021). Instagram, therefore, offers a channel for parents to seek advice and discuss a wide variety of child and food related topics, as well as behavioral information around the practices and practicalities of feeding a family (Saher *et al*. 2024). Furthermore, even if parents are not actively seeking this information, they may still be inadvertently exposed to messages surrounding food, nutrition, and feeding families, particularly if they have engaged with content related to food or family life previously due to the influence of the algorithm (Pariser 2011).

Parents experience common challenges when feeding their children, particularly at family meals where all members of the family are expected to be fed in the same place at the same time (Middleton *et al*. 2023a). As such, parents frequently seek peer and practical support regarding mealtimes (Fraser *et al*. 2021). Feeding children requires consideration of individual food preferences, developmental stages, family resources and logistical practicalities (Middleton *et al*. 2022). Other influences on what and how parents feed their children include cultural customs and social norms. Real and perceived internal and external expectations on what and how children should be fed, and both evidence-based (e.g. national nutrition and infant feeding guidelines) and anecdotal advice (e.g. peer and generational advice) can all play a role (Mehta *et al*. 2020, Middleton *et al*. 2022). This is particularly pertinent in the family meal context, with additional pressures of feeding children “well,” promoting family bonding, while simultaneously providing a pleasant environment and important developmental opportunities. Social media offers a platform for both sharing and searching for information around family mealtimes and Instagram comprises a plethora of accounts and hashtags related to information about food and nutrition. However, while accessible and frequently used for nutrition advice and information, the messages promoted on social media are not always evidence-based, realistic or safe and social media-based misinformation has been identified as a major public health issue (Denniss and Lindberg 2025). Nutrition misinformation has been shown to be prevalent on social media (Denniss *et al*. 2023b), including Instagram, with a recent study finding that 45% of nutrition-related posts by influential accounts contained misinformation, with the highest rates of misinformation related to supplements and children’s nutrition (Denniss *et al*. 2024). This suggests that messaging about family meals may be misleading, and parents may be exposed to information that contradicts evidence-based guidelines and leads to additional stress, pressure, and mental load.

Representation of family meals across general media and health promotion has been criticized as being unrealistic due to portrayals of highly stereotyped, idealized, and “traditional” family mealtimes that are not representative, or achievable for many modern families (Le Moal *et al*. 2025). This representation may contribute to parents feeling guilt, shame, and failure when family meals are challenging or infrequent (Kling *et al*. 2009, Woolhouse *et al*. 2019). These dominant family meal narratives include what foods are prepared (e.g. home-made, balanced, and from-scratch), who prepares them (e.g. a loving caregiver and typically a mother) and how they are consumed (e.g. altogether, around a table, pleasant, instructional, and convivial) (Charles and Kerr 1988, DeVault 1991, Daragan *et al*. 2023). This narrative is unintentionally perpetuated by a focus on correlational benefits to child dietary intake and health with frequent family meals (Dallacker *et al*. 2018), and minimal focus on the quality of the meal environment, the work involved in bringing families together for a meal, or how to address common family meal challenges (Middleton *et al*. 2020). Social media could be an ideal channel for promoting realistic, achievable family meals and sharing practical advice on how to feed families in the context of busy family life. In contrast, as an unregulated platform with no restrictions on who can generate content, it could be providing inappropriate and unrealistic advice and messages about family meals. Research into how family meals are portrayed on social media is currently limited, and evidence regarding who is posting about family meals, the content and what narratives about mealtimes they are sharing with the digital world is lacking.

There is ample evidence surrounding parents’, especially mothers’, perceptions of family meals when asked in a research setting. Parents express that family meals are considered important (Litterbach *et al*. 2017, Middleton *et al*. 2023b) and offer opportunities for child development, optimal nutrition and family connection (Middleton *et al*. 2020). Parents report that family meals can be enjoyable (Daragan *et al*. 2023) but can also be stressful, requiring enormous mental load, organization, and preparation (Middleton *et al*. 2022). Such information has been critical for understanding family meals; however, collecting information this way may not be capturing the inherent social constructs or pressures that surround the family meal. Little is understood about how people talk about and depict the family meal outside of a research environment. Exploring how people discuss family meals in a less structured setting, for example on social media, can provide further insight into the dynamic nature of family meals discussions. Further, understanding the content parents may be exposed to online may provide a new way of understanding realities of family meals and how we can use social media to promote achievable, realistic family meals.

This research set out to explore and better understand how family meals are portrayed on social media. The aims of this study were to: identify who is posting about family meals on Instagram and understand how people are portraying and representing family meals on Instagram.

## METHODS

### Study design

This study used a qualitative content analysis approach to analyze the text-based captions, images and videos of Instagram posts about family meals and the bios and characteristics of the associated Instagram accounts. A descriptive approach to qualitative analysis was used and the research team used a constructivist epistemology (Sandelowski 2000, 2010). Each of the three authors were involved in all aspects of the research, including sample selection, data collection, development of the coding framework and data analysis. All authors are white, cis-gendered women with academic backgrounds in nutrition, are active users of social media, including Instagram, and often engage with social media content related to food and nutrition. EL is a married parent of school-aged children and plans, prepares, and engages in family meals on a weekly basis. ED and GM are married without children and are both actively involved in the planning and preparation of meals for their households. All authors were familiar with the topic of investigation and are thus considered “insiders” (Berger 2015). As white, cis-gendered women with academic backgrounds in health and nutrition, the researchers acknowledge that their perspectives and beliefs may have influenced interpretation of the data. The research team met regularly throughout the planning, data collection and analysis phases to discuss and critically evaluate their interpretation of the data, to reduce the risk of potential biases.

This research was undertaken in accordance with the Declaration of Helsinki. The dataset was comprised of publicly available information, and therefore waiver of consent was sought and approved by Flinders University Human Ethics Committee (#6860). We sampled hashtags for posts and collected available information on public accounts (e.g. bio and number of followers). The research team did not interact with any posts or users through the course of data collection. Any quotes of text captured through data collection have been searched in Google to ensure they do not link back to account holders (Denniss *et al*. 2023a, 2023b). All data were managed and stored in a secure password protected Research Electronic Data Capture (REDCap) database (Harris *et al*. 2009, Harris *et al*. 2019).

### Sample selection

At the time this research was conducted it was not possible to systematically identify the “top” posts (i.e. most popular posts) on a specific topic via keyword searches in Instagram’s search engine. This is due to the influence of Instagram’s algorithm and the restriction of access to Instagram’s Application Programming Interface. However, searching for a specific hashtag in Instagram’s search engine will return the “top” posts that have included the hashtag. To enable the systematic identification of popular posts to include in this study, posts were identified via searching hashtags relevant to family meals. Use of hashtags for sample selection is a common approach in studies that use Instagram posts as the data source (e.g. Tiggemann and Zaccardo 2018, Jebeile *et al*. 2021, Hoare *et al*. 2022).

**Figure 1.**
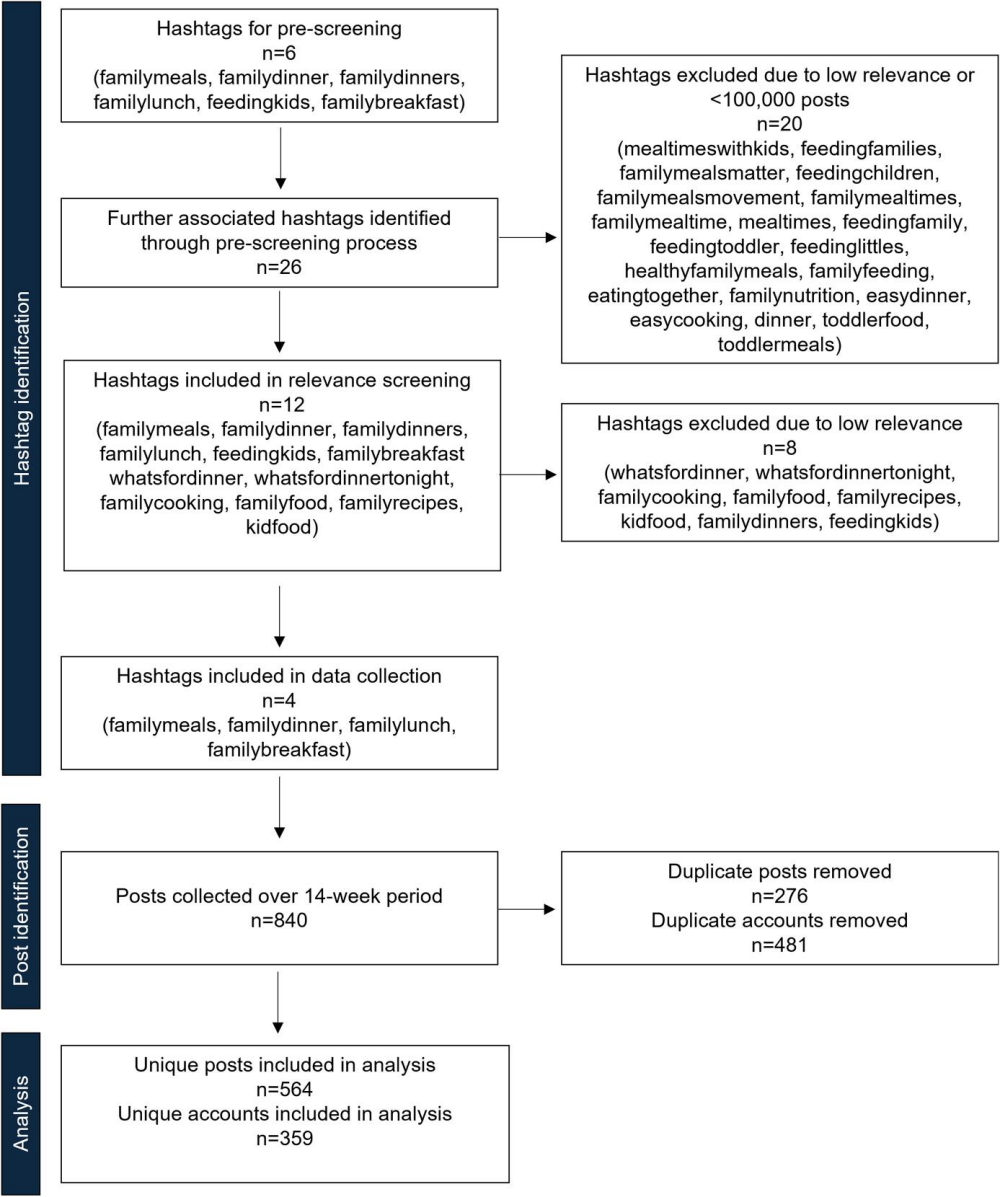
Flow-chart of hashtag selection and postcollection process.

To identify relevant hashtags, the team identified six hashtags to prescreen: #familymeals, #familydinner, #familydinners, #familylunch, #feedingkids, and #familybreakfast based on a preliminary exploration of Instagram. All of these hashtags had >100 000 posts in total, thus were deemed popular enough for inclusion in prescreening. The research team then reviewed posts that had been tagged with these hashtags to identify additional hashtags for screening. A total of 32 hashtags were identified as potentially relevant. After review, 20 hashtags were excluded due to low relevance of posts (content not relevant to family meals, mealtimes, meal planning, meal preparation, food, or feeding families) or low overall number of posts (<100 000 posts tagged, with 100 000 posts chosen as a cut point because hashtags with 10 000–200 000 posts are considered popular yet niche; Demeku 2025). Twelve hashtags were included in the screening process, where the top nine posts of each hashtag were checked every second day for 14 days to determine post relevance to family mealtimes and turnover (how frequently the top nine changed). A Google Chrome browser in “Incognito” mode and new Instagram accounts without user data were used for hashtag screening, to minimize the likelihood of Instagram’s algorithm influencing the search results. Eight hashtags were excluded due to low relevance of posts, low overall number of posts, or low frequency of posts (top nine posts did not change over the 2-week period). The four remaining hashtags #familymeals, #familydinner, #familylunch, and #familybreakfast were included in this study, as they met inclusion criteria (number of posts, frequency of posts, relevance of posts), and were deemed the most likely hashtags parents would search for if they were looking for content on family meals. Figure 1 depicts a flow-chart of the sample selection process.

### Data collection

The four selected hashtags #familymeals, #familydinner, #familylunch, and #familybreakfast were visited once a week for 14 weeks from 15 February to 16 May 2024. This period of time was chosen as it did not coincide with a major food holiday (e.g. Thanksgiving, Halloween, and Christmas), but did include some periods of celebration (e.g. Easter and Mother’s Day). The “top” 15 posts of each hashtag were collected, totaling 60 posts per week and 840 posts throughout the data collection period. This number was chosen to account for the collection of duplicate or irrelevant posts and because sample sizes of ∼600 posts are common in Instagram content analyses (e.g. Tiggemann and Zaccardo 2018, Cohen *et al*. 2019, Denniss *et al*. 2024). Permanent “grid” posts, including Reels, single image posts and carousels (posts with multiple images) were collected and Instagram “stories,” which are temporary and only visible for 24 h, were not collected. Stories were not collected due to their temporary nature and because they are not visible when searching for “top” posts via Instagram’s search function.

Data were collected by manually downloading the post image and/or video content and populating a purpose-built REDCap database with information about the account (handle, bio, and number of followers), and the post (caption, likes, number of comments, and format). Data were collected manually to comply with Instagram’s terms of service, which prohibit data scraping, and to comply with Australian research ethics standards, which specify that researchers must comply with the terms and conditions set out by social media platforms (National Health and Medical Research Council *et al*. 2023). One member of the research team was responsible for one or two hashtags and hashtags were swapped between researchers across several weeks of data collection to ensure researcher exposure to each hashtag. For consistency, data were collected on the same day each week (Thursday), which was chosen based on the availability of the researchers.

### Coding framework development

To the knowledge of the authors, a framework relevant to family mealtimes on social media has not previously been developed. As such, after data collection was complete the team collaboratively developed a preliminary coding framework using an inductive approach. All members of the research team were immersed in the data due to their exposure to Instagram posts throughout the 14-week data collection period. The framework had three sections. The first section included codes for the Instagram accounts, the second included codes for the text-based captions and the third section contained codes for the visual elements of posts (images and videos). This approach, whereby accounts, captions and visual elements are coded or classified separately, was informed by previous social media content analysis research (Tiggemann and Zaccardo 2018, Cohen *et al*. 2019, Hoare *et al*. 2022, Denniss *et al*. 2023a). EL tested the preliminary framework using a subsample of accounts and posts from each hashtag (*n* = 60) and inductively adapted the framework as necessary. Before coding of the dataset commenced, the research team held three collaborative workshops (5 h in total) to thoroughly test and adapt the coding framework using a subsample of posts from each hashtag to ensure the framework was interpreted consistently by the team. The coding framework is included in Supplementary Tables S1 and S2.

### Analysis

After data collection had concluded, duplicate Instagram accounts and posts were excluded from the dataset for each hashtag. If an account or post was captured under multiple hashtags, one observation for each of the hashtags was included in analysis (i.e. duplicate accounts and posts were kept in the sample once under each hashtag they were associated with). When an account or post appeared in duplicate, the first datapoint was included and subsequent observations were excluded from analysis.

After testing and finalizing the framework, coding was undertaken independently by all members of the research team in REDCap. When applying codes for the included Instagram accounts, the bio at the time of data collection was considered and the public profile for each account was also visited to assist with determining the most frequent type of content posted (e.g. recipes, parenting advice, and health information). Accounts were classified as one of the following categories, either by self-identification in their bio or by the research team: recipe developer, food blog, parenting blog, dietitian/nutritionist, weight loss focus, public figure, or general account. Where information was available, gender of the account holder was classified either through self-identification in their bio (woman, man, and nonbinary), or through visual presentation and depiction aligning with typical gendered representations of feminine (woman) or masculine (man). Parenting status was also classified in a similar way, through what was stated in the account bio where available, or through what was depicted in posts. This was done solely with the intention of exploring if the gendered divide of food work is or is not represented and perpetuated through social media. For all codes that were applied for accounts, it was also recorded whether the code applied was self-identified by the account holder explicitly stating it on their profile or if it was identified based on the researcher’s interpretation of their profile (e.g. self-identified as a food blogger, appears as a mother).

When coding posts, the original post on Instagram was viewed so it could be considered in the format and context in which Instagram users engage with it. If posts had been deleted or were no longer visible on the platform, the extracted data in the database was used for coding (saved images, videos, and caption). Post captions were read in full, and codes were applied to classify the written information (e.g. meal ideas and information about food planning). All video and image content for a post was considered when coding the visual elements and descriptive codes were applied (e.g. food depicted and people depicted). When food was depicted it was also coded as core foods or discretionary foods according to the Australian Guide to Healthy eating (Australian Government *et al*. 2013) and based on the majority of the food pictured in the post (e.g. if both discretionary and core foods were depicted, a judgment was made based on which category was the most visually represented in the image or video). Accounts or posts that were ambiguous or difficult to code were flagged for team discussion to reach consensus. Once coding in REDCap was complete, data were exported to Stata/SE 17 (StataCorp, College Station, TX) and descriptive statistics were run. It is common practice to only report descriptive statistics in content analysis studies (e.g. Hoare *et al*. 2022, Denniss *et al*. 2023a) and therefore, no inferential statistical tests were run to assess significance of differences between hashtags.

## RESULTS

### Accounts

Across all hashtags, there were posts from 359 unique accounts. Characteristics of included accounts are summarized in Table 1. Accounts had a median of 15 600 followers, majority were a single-account holder, with a small proportion of group accounts (18.4%). Majority of accounts were self-identified or classified as recipe developers (38.4%). Weight loss, public figure and dietitian/nutrition accounts were the least common across all hashtags (2.2%–2.8%). Of those accounts where gender was self-identified or classified (249 accounts), majority were women (86.3%). Of accounts where parenting status was self-identified or could be classified based on post content (138 accounts), 95.7% were mothers.

**Table 1.** Characteristics of unique Instagram accounts captured in the content analysis.

Account category	All*n* (%)	Followers (all accounts)Median ± IQR	#familybreakfast*n* (%)	#familylunch*n* (%)	#familydinner*n* (%)	#familymeals*n* (%)
Total	359	15 600 ± 87 661	98	108	89	64
Account held by individual	293 (81.6)	2 789 ± 44 556	79 (80.6)	84 (77.7)	76 (85.4)	54 (84.4)
Account held by group	66 (18.4)	19 700 ± 91 454	19 (19.3)	24 (22.2)	13 (14.6)	10 (15.6)
Account type^a^						
Recipe developer	138 (38.4)	37 200 ± 165 413	36 (36.7)	17 (15.7)	45 (50.6)	40 (62.5)
Self-identifies as	92 (66.7)	–	26 (72.2)	10 (58.8)	27 (60.0)	29 (72.5)
Appears as	46 (33.3)	–	19 (27.8)	7 (41.2)	18 (40.0)	11 (27.5)
Food blog	77 (21.4)	1 831 ± 19 989	14 (14.3)	37 (34.3)	13 (14.6)	13 (20.3)
Self-identifies as	32 (41.6)	–	8 (57.1)	17 (46.0)	6 (46.2)	1 (7.7)
Appears as	45 (58.4)	–	6 (42.9)	20 (54.0)	7 (53.8)	12 (92.3)
General	80 (22.3)	3 004 ± 39 538	21 (21.4)	44 (40.7)	15 (16.9)	–
Self-identifies as	26 (32.5)	–	6 (28.6)	17 (38.6)	3 (20.0)	–
Appears as	54 (67.5)	–	15 (71.4)	27 (61.4)	12 (80.0)	–
Parenting blog	32 (8.9)	37 050 ± 78 132	17 (17.3)	4 (3.7)	7 (7.9)	4 (6.3)
Self-identifies as	15 (46.9)	–	7 (41.2)	3 (75.0)	3 (42.9)	2 (50.0)
Appears as	17 (53.1)	–	10 (58.8)	1 (25.0)	4 (57.1)	2 (50.0)
Dietitian/nutritionist^b^	10 (2.8)	28 900 ± 374 158	3 (3.1)	–	3 (3.4)	4 (6.3)
Weight loss	9 (2.5)	64 500 ± 37 500	2 (2.0)	1 (0.9)	4 (4.5)	2 (3.1)
Self-identifies as	8 (88.9)	–	1 (50.0)	1 (100.0)	4 (100.0)	2 (100.0)
Appears as	1 (11.1)	–	1 (50.0)	–	–	–
Public figure	8 (2.2)	348 500 ± 506 050	4 (4.1)	2 (1.9)	2 (2.2)	–
Self-identifies as	7 (87.5)	–	4 (100.0)	1 (50.0)	2 (100.0)	–
Appears as	1 (12.5)	–	0 (0)	1 (50.0)	–	–
Gender^a,c^	**–**	**–**	**–**	**–**	**–**	**–**
Woman	215 (73.4)	28 600 ± 107 723	63 (79.7)	54 (64.3)	62 (81.6)	52 (96.3)
Self-identifies as	6.9 (16)	–	2 (3.2)	3 (5.6)	5 (8.1)	6 (11.5)
Appears as	93.1 (215)	–	61 (96.8)	51 (94.4)	57 (91.9)	46 (88.5)
Man	32 (7.8)	4554 ± 35 853	5 (6.3)	16 (19.0)	9 (11.8)	2 (3.7)
Self-identifies as	1 (3.1)	–	–	1 (6.2)	–	–
Appears as	31 (96.9)	–	5 (100.0)	15 (93.8)	9 (100.0)	2 (100.0)
Nonbinary	2 (0.7)	195 ± 44	2	–	–	–
Self-identifies as	2 (100.0)	–	2 (100.0)	–	–	–
Parenting^a,c^						
Mother	132 (45.0)	24 650 ± 96 230	42 (53.2)	21 (25.0)	38 (50.0)	31 (57.4)
Self-identifies as	89 (67.4)	–	23 (54.8)	16 (76.2)	26 (68.4)	24 (77.4)
Appears as	43 (32.6)	–	19 (45.2)	5 (23.8)	12 (31.6)	7 (22.6)
Father	6 (2.0)	3 705 ± 27 721	–	3 (3.6)	3 (3.9)	–
Self-identifies as	3 (50.0)	–	–	1 (33.3)	2 (66.8)	–
Appears as	3 (50.0)	–	–	2 (66.7)	1 (33.3)	–

^a^Less than 100% of total *n* because the characteristic could not be determined for some accounts due to it being unclear or the account being deleted or made private after data collection.

^b^No option for “appears as” account holder must have identified as the relevant code in their bio.

^c^Percentage calculated based on number of accounts held by individuals (*n* = 293).

### Differences across hashtags

The hashtag #familylunch had the most unique accounts (*n* = 108), compared to #familymeals, which had the least (*n* = 64). Individual accounts were predominant across all hashtags, with #familylunch having the largest proportion of group accounts (22.2%). Accounts classified as recipe developers constituted the largest proportion of account categories across all hashtags except for #familylunch, which had a higher proportion of accounts self-identified or classified as food blog (34.3%) or a general account (e.g. normal person, lifestyle influencer, and travel influencer) (40.7%). The hashtag #familybreakfast had the highest proportion of self-identified or classified parenting blogs (17.3%), and #familylunch had the highest proportion of accounts classified as belonging to men. Only #familylunch and #familydinner had accounts classified as fathers, consisting of <5% of all individual accounts in each.

### Posts

As shown in Table 2, there were 564 unique posts identified across all hashtags, after removal of duplicates. Posts were almost evenly split between carousel (29%), single image (33.3%) and single video posts (36.7%). Engagement with posts across all hashtags was moderate, with median *n* = 307 ± 1 559 likes and median *n* = 21.5 ± 73 comments on each post.

**Table 2.** Number of unique posts captured under each hashtag and engagement (likes and comments).

Hashtag	Unique posts *n*	Carousel posts *n* (%)	Single image posts *n* (%)	Single video posts *n* (%)	Post likes median ± IQR	Comments median ± IQR
All	564	169 (29.0)	188 (33.3)	207 (36.7)	307 ± 1 559	21.5 ± 73
#familybreakfast	121	26 (21.5)	51 (42.2)	44 (36.4)	100 ± 849	3 ± 28
#familylunch	127	64 (50.4)	30 (23.6)	33 (26.0)	49 ± 698	2 ± 16
#familydinner	152	57 (37.5)	30 (19.7)	65 (42.8)	1 177.5 ± 2 853	37 ± 84.5
#familymeals	164	22 (13.4)	77 (47.0)	65 (39.6)	310 ± 1 665	52.5 ± 153.5

Table 3 presents the post content across the hashtags. Majority of posts across all hashtags depicted food/drink (92.9%), mostly plated food (86.6%) and few restaurant meals (10.1%), with modest representation of cooking (41.6%) or recipe information (31.7%). Posts depicting food/drink predominantly consisted of core foods (76.7%) compared to discretionary foods (23.3%). Almost two-thirds of posts were classified as appearing staged or altered to conform with desired aesthetics (64.7%). Less than 15% of posts depicted sharing a meal together (11.3%), where they did, majority were shared outside the home (64.1%). People were depicted in 137 of posts (26.1%), excluding posts where only the hands and torso were depicted when preparing food. Where people were depicted, it was typically multiple people (56.2%), with most posts including adults (79.6%) and women (75.9%). Men were depicted in 37.2% of posts, children were depicted in 43.8% of posts and less than a third of posts included families (30.7%).

**Table 3.** Codes for images/videos for unique posts captured in the content analysis.

Visual element	All (*n* = 564)*n* (%)	#familybreakfast (*n* = 121)*n* (%)	#familylunch (*n* = 127)*n* (%)	#familydinner (*n* = 152)*n* (%)	#familymeals (*n* = 164)*n* (%)
Food/drink^a^	524 (92.9)	115 (95.0)	105 (82.7)	143 (94.1)	161 (98.2)
Plated food	453 (86.6)	93 (80.9)	85 (81.0)	130 (90.9)	145 (90.1)
Cooking	218 (41.6)	42 (36.5)	15 (14.3)	97 (67.8)	64 (39.8)
Recipe	166 (31.7)	24 (20.9)	5 (4.8)	79 (55.2)	58 (36.0)
Restaurant meal	53 (10.1)	7 (6.1)	39 (37.1)	7 (4.9)	–
Discretionary food	122 (23.3)	51 (44.4)	47 (44.8)	6 (4.2)	18 (11.2)
Core food	402 (76.7)	64 (55.7)	58 (55.2)	137 (95.8)	143 (88.8)
Sharing a meal^a^	64 (11.3)	10 (8.3)	39 (30.7)	14 (9.2)	1 (0.6)
Outside the home	41 (64.1)	4 (40.0)	30 (76.9)	7 (50.0)	–
At home	20 (31.3)	6 (60.0)	6 (15.4)	7 (50.0)	1 (100.0)
Engaging in celebration	4 (6.3)	–	4 (10.3)	–	–
People depicted	137 (26.1)	24 (19.8)	52 (40.9)	44 (28.9)	17 (10.4)
Adult	109 (79.6)	19 (79.2)	45 (86.5)	32 (72.7)	13 (76.5)
Woman	104 (75.9)	18 (75.0)	43 (82.7)	30 (68.2)	13 (76.5)
Multiple people	77 (56.2)	16 (66.3)	39 (75.0)	15 (34.1)	7 (41.2)
Child/children	60 (43.8)	15 (62.5)	26 (50.0)	11 (25.0)	8 (47.1)
Man	51 (37.2)	6 (25.0)	35 (67.3)	8 (18.8)	2 (11.8)
One person	48 (35.0)	8 (33.3)	8 (15.4)	22 (50.0)	10 (58.8)
Family	42 (30.7)	8 (33.3)	24 (46.2)	8 (18.2)	2 (11.8)
Tablescape	26 (4.6)	5 (4.1)	18 (14.2)	3 (2.0)	–
Appears staged	365 (64.7)	80 (66.1)	56 (44.1)	96 (63.2)	133 (81.1)
Appears authentic	176 (31.2)	36 (29.8)	59 (46.5)	52 (34.2)	29 (17.7)
Irrelevant	23 (4.1)	5 (4.1)	12 (9.4)	4 (2.6)	2 (1.2)

^a^More than one subcode could be selected for posts coded under the parent code.

Table 4 provides post caption content across the hashtags. Less than one fifth of captions across hashtags were not in English or not relevant (18.4%). Most hashtags included meal ideas (70.6%) that linked out to recipes (40%), for example: “**To Get the Recipe:**  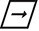 Tap the link in my bio…”—#familydinner, 3027, or provided recipes directly in caption (38.4%).

**Table 4.** Codes for captions of unique posts captured in the content analysis.

Caption	All (*n* = 564)*n* (%)	#familybreakfast (*n* = 121)*n* (%)	#familylunch (*n* = 127)*n* (%)	#familydinner (*n* = 152)*n* (%)	#familymeals (*n* = 164)*n* (%)
Meal ideas^a^	398 (70.6)	84 (69.4)	34 (26.8)	125 (82.2)	155 (94.5)
Recipe elsewhere	159 (40.0)	11 (13.1)	5 (14.7)	66 (52.8)	77 (49.7)
Quick/easy	155 (38.9)	25 (29.8)	7 (20.6)	52 (41.6)	71 (45.8)
Recipe in post	153 (38.4)	47 (56.0)	8 (23.5)	39 (31.2)	59 (38.1)
Kid/family friendly	99 (24.9)	28 (33.3)	5 (14.7)	26 (20.8)	40 (25.8)
Comfort food	33 (8.3)	4 (4.8)	3 (8.8)	11 (8.8)	15 (9.7)
Healthy	30 (7.5)	12 (14.3)	–	9 (7.2)	9 (5.8)
Budget friendly	18 (4.5)	3 (3.6)	2 (5.9)	12 (9.6)	1 (0.65)
Freezer friendly	17 (4.3)	4 (4.8)	–	9 (7.2)	4 (2.6)
“Healthy” alternative	17 (4.3)	7 (8.3)	–	7 (5.6)	3 (1.9)
Fussy eater friendly	6 (1.5)	–	–	2 (1.6)	4 (2.6)
Food descriptions^a^	160 (28.4)	18 (14.9)	16 (12.6)	67 (44.1)	59 (36.0)
Taste	132 (82.5)	14 (77.8)	14 (87.5)	59 (88.1)	45 (76.3)
Texture	82 (51.3)	6 (33.3)	4 (25.0)	34 (50.8)	38 (64.4)
Aesthetic	12 (7.5)	–	4 (25.0)	6 (9.0)	2 (3.4)
Smell	7 (4.4)	1 (5.6)	1 (6.3)	2 (3.0)	3 (5.1)
Food planning^a^	21 (3.7)	4 (3.3)	1 (0.8)	4 (2.6)	12 (7.3)
Meal planning bulk	13 (61.9)	3 (75.0)	1 (100.0)	3 (75.0)	6 (50.0)
Cooking	12 (57.1)	3 (75.0)	–	1 (25.0)	8 (66.7)
Shopping lists	6 (28.6)	2 (50.0)	–	2 (50.0)	2 (16.7)
Celebration	25 (4.4)	3 (2.5)	17 (13.4)	4 (2.6)	1 (0.6)
Tradition	10 (1.8)	1 (0.8)	3 (2.4)	1 (0.7)	5 (3.0)
Experience of mealtimes	42 (7.4)	16 (13.2)	10 (7.9)	5 (3.3)	11 (6.7)
Emotions/feelings	40 (7.1)	7 (5.8)	21 (16.5)	12 (7.9)	–
Information/advice	27 (4.8)	6 (5.0)	1 (0.8)	4 (2.6)	16 (9.8)
Not relevant/not in English	104 (18.4)	26 (21.5)	58 (45.7)	16 (10.5)	4 (2.4)

^a^More than one subcode could be selected for posts coded under the parent code.

Over one third of captions included phrases like “quick” or “easy” (38.9%) and just under one quarter used words indicating foods were kid or family friendly (24.9%).

EASY family breakfast ideas Here are a few more healthy breakfast ideas for the week ahead the whole family will enjoy. My favourite is the PB&J French Toast, which one would you make first? …—#familybreakfast, 1011

Whipping up a scrumptious lunch in a snap! Quick, easy, and oh-so-delicious—perfect for sharing with the whole family. Let's dig in! 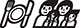
…—#familylunch, 2149

Kid Friendly [curry recipe]…literally the easiest, most kid friendly recipe and what toddler doesn't love bread 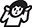
… #familydinner, 3113

If you're in search of a simple and family-friendly [removed] recipe, I've got you covered! It's super easy to make, and you can whip up this dish in under 30 minutes…—#familylunch, 2151

Few post captions included reference to comfort food (8.3%), healthy food (7.5%), freezer friendly, or “healthy” alternatives (both 4.3%), or reference to suitability for fussy eaters (1.5%).

This Sloppy Joe Casserole recipe has everything you love about the classic sauce sandwiches, but in the form of a pasta casserole instead! Simple to make, hearty, and filling, this is a warm and cozy meal that everyone will love! …—#familymeals, 4205

Here's a really easy breakfast (or snack) recipe for you and your little ones. You only need a handful of ingredients for a delicious healthy French toast recipe with no added sugar…—#familybreakfast,1031

…Meat consumption tends to be one of the top battles for parents with picky eaters this is due to the textures that come with it, we have found when using minced meats and forming them into easy to grip shapes they ease anxiety and are more likely to be enjoyed…—#familymeals, 4137

Over a quarter of post captions included descriptions of food (28.4%), predominantly describing the taste (82.5%), followed by texture (51.3%), aesthetic (7.5%), and smell (4.4%).

…wonderfully juicy, sweet and spicy … crispy SLAW and creamified……A flavor and texture explosion…!—#familydinner, 3081

Beautifully roasted chicken in a garlicky, lemony marinade sits on a bed of potatoes that soak up all of the goodness and become soft and caramelised. It is insanely delicious…—#familydinner, 3031

…I love the flavours and textures in this, crisping up the gnocchi makes all the difference ∼ all wrapped up in a velvety sauce 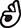
… -#familymeals, 4020

Food planning was rarely mentioned in post captions across hashtags (3.7%), but where it was mentioned, majority referenced meal planning (61.9%) or bulk cooking practices (57.1%).

…The perfect recipe for batch cooking and to get you back on plan…Cooking it in the slow cooker means you can chuck everything in and walk away and get on with your day… it freezes well too 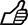
…—#familymeals, 4003

### Differences across hashtags

The hashtag #familymeals comprised 164 unique posts, the highest across the hashtags, and #familybreakfast comprised the fewest at 121. Comparing across the hashtags, carousel posts were most common for #familylunch (50.4%), single image posts were most common for #familymeals (47%) and single video posts were most common for #familydinner (42.8%). Engagement with posts was lowest for #familylunch (median 49 ± 698 likes and median 2 ± 16 comments) and highest for #familydinner (median 1177.5 ± 2853 likes and median 37 ± 84.5 comments).

Food/drink was highly depicted across all hashtags, but #familydinner contained the highest proportion of representations of cooking (67.8%) and recipe information (55.2%). The hashtag #familylunch depicted the highest proportion of meals at restaurants (37.1%) compared to #familymeals where no restaurant meals were depicted. Both #familybreakfast and #familylunch contained the highest proportion of depictions of discretionary foods (44.4% and 44.8%, respectively), and #familydinner contained the highest proportion of core foods (95.8%) and lowest of discretionary foods (4.2%). The hashtag #familylunch depicted the highest proportion of people sharing a meal together (30.7%), typically outside the home (76.9%). Accordingly, #familylunch also depicted the highest proportion of multiple people in posts (40.9%), adults (86.5%), men (67.3%), and families (46.2%). Only #familybreakfast had a higher depiction of children (62.5%) than #familylunch (50%). Conversely, #familymeals contained the fewest posts depicting people sharing a meal together (0.6%) and the fewest depictions of families (11.8%). The highest proportion of posts classified as appearing staged was found in #familymeals (81.1%), and #familylunch had the highest proportion of posts appearing authentic (46.5%).

The hashtag #familylunch contained the most captions that were not in English or not relevant (45.7%). Captions in #familybreakfast included the highest proportion of mentions of “healthy foods” (14.3%), “healthy alternatives” (8.3%), kid or family friendly meals (33.3%), and contained the highest proportion of recipes in post (56%).

…FAKE-AWAY FOR THE FAMILY … SAVE MONEY AND SAVE YOUR CALORIES! …—#familybreakfast, 1009

… just 4 ingredients is all you need to make these delicious little breakfast pastries, that really taste and feel like an indulgent feast, with no added sugar and especially none of the guilt!—#familybreakfast, 1029

…What should I give my kids for breakfast? … We want it to be fast, easy, and nutritious. Here are 8 of my go-to breakfasts …all either quick to make or can be made ahead, and most importantly kid approved!”—#familybreakfast, 1019

The hashtag #familymeals included the highest proportion of reference to comfort foods (9.7%), quick and or easy meals (45.8%).

…Delicious, quick and comforting chicken noodle soup, ready in 15 minutes! ….—#familymeals, 4062

Reference to freezer friendly and budget friendly meals were highest in #familydinner (7.2% and 9.6%, respectively), as were captions that linked out to recipes hosted elsewhere (52.8%).

…You can also assemble the whole thing and bake it up to 2 days later or freeze it and bake it months later…—#familydinner, 3206

…if you need to make money stretch, this [removed] pasta is delicious and kid friendly 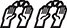
—#familydinner, 3007

The #familydinner hashtag also contained the highest proportion of posts describing food (44.1%).

…the perfect blend of simplicity and flavor, …There's nothing like serving this with a side of creamy mashed potatoes and a fresh, easy tossed salad….simple ingredients, amazing flavors, and happy faces around the dinner table. …—#familydinner,3178

Food planning was mentioned most frequently in #familymeals captions (7.3%), and this hashtag contained the highest proportion of reference to information or advice (9.8%) and to tradition, although still low at 3.0%.

…Here's how I do it: 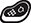 Only buy meat on sale and freeze it 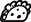 Plan meals around what meat I have for two weeks 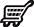 Shop at an affordable grocery store and compare prices between stores 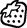
Shop in-season produce 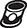 Buy extra shelf stable items when they're on sale (only buy ones that you will actually use) 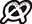
Don't buy prepackaged snacks 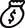
I've been shopping this way for over 5 years now and it has saved us tens of thousands of dollars!—#familymeals, 4104

…We cook this twist on a traditional Irish meal every St. Patrick's Day and it's become a favorite family tradition
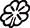
—#familymeals, 4077

Reference to celebrations, emotions and/or feelings were highest in #familylunch captions (13.4% and 16.5%, respectively), but #familybreakfast contained the highest proportion of reference to mealtime experience (13.2%).

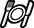

Lunchtime Adventures with My Little Ones! 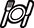 Spent a lovely afternoon having lunch with my favorite people. The smiles, giggles, and yummy food made it a perfect day! 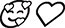
—#familylunch, 2207

Heartwarming Breakfast Bonding: Family Moments Gathered around the breakfast table, this family shares spontaneous moments filled with love and affection. From laughter to heartfelt hugs, their bond shines bright. 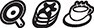
…—#familybreakfast, 1095

Anyone else out there constantly stressed about what they are going to serve for dinner? Honestly, family meals are the bane of my existence. I am constantly struggling to find easy, healthy meals my entire family will eat…Is cooking a source of stress for you or do you love it?—#familymeals, 4028

## DISCUSSION

This study aimed to explore how family meals are represented and portrayed on Instagram. Through systematically collecting top posts from four hashtags and applying a content analysis approach, we analyzed 564 unique posts and 359 unique accounts. Our findings demonstrated that majority of users posting content about family meals are recipe developers who are predominantly women or mothers, and most posts depict highly curated and plated food or drink. With very few “authentic” posts of families eating together, our analysis suggests there is a stark divide between social media representations of family meals and the real-life experiences of parents.

Currently, there is no agreed upon definition of what constitutes a “family meal,” where or when it takes place, who is there, what food is being consumed and who is preparing it (Daragan *et al*. 2023, Snuggs and Harvey 2023). However, there is a common Western understanding that family meals occur in the evening and consist of a warm, nutritious, home-cooked meal prepared by a devoted caregiver, usually a mother (Charles and Kerr 1988, DeVault 1991, James *et al*. 2009, Bowen *et al*. 2019, Woolhouse *et al*. 2019, Oleschuk 2020, Daragan *et al*. 2023). This differs from the daily experiences of many families, with numerous barriers preventing families from sharing meals together in such an idealized way (Mehta *et al*. 2020, Woolhouse *et al*. 2019, Middleton *et al*. 2023a). However, the notion of the idyllic family meal holds consistent across social media, with most of the posts captured in this study appearing polished, staged, and curated. There were few posts across hashtags of families eating a meal together in their home, with most posts depicting clean kitchens, pristine counter tops, high-quality cooking equipment, beautifully plated dishes, and decorated tablescapes. These curated images and videos may be reinforcing unrealistic expectations and unattainable standards for families who are seeking advice, tips or guidance for family meals in their household. Literature already posits that unrealistic expectations on the ideal family meal perpetuated by social norms, in media and health promotion lead to feelings of guilt, failure and shame when unable to live up to them (Oleschuk 2020, Le Moal *et al*. 2021). While the curated nature of social media is well known, the pervasive and frequent comparison provided by such platforms can have a profound impact on an individual’s confidence, self-esteem and mental wellbeing (Prichard *et al*. 2020, Wirtz *et al*. 2021, Han and Yang 2023, Rosenthal and Tobin 2023, Wu *et al*. 2024). This suggests that the highly staged and performative nature of these posts may make parents feel less capable of preparing family meals, rather than giving them the skills and motivation to do so.

Further contributing to the perpetuation of potentially harmful narratives was the high proportion of mothers and women across accounts and depicted in posts. While men constitute over 50% of Instagram users (Statista 2024), the greater proportion of women depicted in the present study is concerning regarding perpetuation of gendered stereotypes and the nature of food work. Traditionally the provision of food for the family has been considered the responsibility of mothers and women because historically men were primarily responsible for generating income while women managed the household (Murcott 1995, Charles and Kerr 1988, DeVault 1991). However, in most Western countries these trends have changed, and women and mothers are engaging in paid employment at increasing rates. With higher proportions of single-parent and dual-employed families, there is no longer a dedicated household manager whose full-time job includes food provision. However, in many households women are still undertaking the majority of household labor in addition to working outside the home (Storz *et al*. 2022). Food provision remains the primary responsibility of mothers and women, likely due to gendered expectations, role-modelling and learned skills (Eagly and Wood 2016, Lupton 2000, Burnod *et al*. 2022). The perpetuation of these stereotypes on social media, while not surprising, is concerning. To shift the burden of responsibility from mothers and women, social norms around who is expected to do this work must shift. This can be facilitated through representation of men and fathers undertaking this type of work and shouldering more of this responsibility. Greater representation of men and father’s cooking and feeding their family on social media may help shift this narrative.

The inclusion of four unique hashtags provided opportunity to scrutinize the social norms around family meals and what they should look like depending on when they occur. The hashtags #familymeal and #familydinner focused primarily on the food rather than the “family” component of the meal. Posts under these hashtags contained the highest proportion of “core” foods, recipe and cooking content that appeared “staged,” and presented a narrative that these meals are about beautifully prepared meals that are healthy, affordable, quick, and easy. Conversely, #familylunch portrayed more authentic images of families eating together, usually outside of the home, implying that family lunches are for connection, togetherness, and celebration. This differed to #familybreakfast, which focused on the experience of the meal rather than the nutritional content. Literature suggests that evening meals are the most frequent shared meals in Western households, as this is typically when families reconvene for the day (Litterbach *et al*. 2017). Typical Western meal patterns also favor the evening meal as the “main” meal, and subsequently the most nutritious meal of the day (Leech *et al*. 2015, Fayet-Moore *et al*. 2020). The social media narratives identified through our analysis reinforce this pressure on evening meals to provide essential nutrition, potentially at the expense of enjoyment or family connection. Conversely, focusing on the meal experience and family connection at other meals may override considerations for nutritional quality. Other cultures do not necessarily have this same categorization of mealtimes, what they consist of or how they should look dependent on the time of day they occur (Fischler 2011, Momin *et al*. 2014, Zirari 2020, Le Moal *et al*. 2021). Social media could be a useful tool for shifting these mealtime norms. For example, by reducing pressure on the evening meal carrying the burden of nutritional quality for the day, we may be able to foster more connection and enjoyment at these meals. Promoting the inclusion of more nutritious foods throughout the day, such as incorporating vegetables and lean protein sources at morning and midday meals, as well as snacks, may help ease this pressure in the evening while also improving nutritional intake across the day.

Instagram posts captured in this study frequently contained meal ideas and recipes. This finding is consistent with other studies that have examined food and nutrition-related content on Instagram and shown that influential accounts frequently post meal ideas and recipes (de Jesus Oliveira *et al*. 2019, Denniss *et al*. 2023a) and recipes often appear alongside healthy eating hashtags (Pilař *et al*. 2021). In the present study, core foods were depicted more frequently than discretionary foods in general and within posts containing recipes and meal ideas. Similarly, Denniss *et al*. (2023a) found that recipes on Instagram often contained fruit, vegetables, lentils and wholegrains as key ingredients. Home cooking is associated with diet quality (Mills *et al*. 2017) and the prominence of “quick and easy” meal ideas and recipes that contain core foods suggests that Instagram may be a useful resource to promote home cooking and improve nutritional intake. There is also evidence that the public use social media to source recipes (Nelson and Fleming 2019, Tricas-Vidal *et al*. 2022), further indicating that it is an accessible and relevant channel to help families plan and prepare healthy meals. However, more research is needed to understand the impact of social media as a nutrition promotion tool for families because nutrition misinformation is common on social media (Denniss *et al*. 2023b, 2024) and findings from this study suggest that family meal content may perpetuate unrealistic standards and gender norms. Furthermore, there is a lack of research on the healthfulness and nutritional quality of recipes from Instagram and other social media platforms and this is a key area for future research.

### Strengths and considerations

This research has its strengths in the systematic identification and selection of hashtags for inclusion in data collection, and the rigorous processes followed for the 3-month period of data collection. Additionally, the coding framework was inductively and iteratively developed and thoroughly tested by all members of the research team, and all researchers responsible for analysis had an active role in data collection and were thus immersed in the content. Being able to capture depictions of family meals from the public, and not from participants registered into a “family meal” study, reduces the social-desirability and self-selection bias common in this type of research, allowing us a window into the “real world” of family meal narratives.

The influence of the algorithm and plethora of content published on Instagram hinder the ability to comprehensively gather posts on a particular topic. While we used top posts in selected hashtags to mitigate this, it is likely that relevant and popular posts regarding family meals that did not use one of the hashtags were missed. Further, gender and parent-status were assigned based on visual assumption where not identified by the account holder, which should be taken into consideration when interpreting results. Finally, discretionary and core foods were coded based on appearance in images/videos and were assigned depending on the proportions of the foods contained in the image, e.g. a meal that contained a protein, vegetables and fried chips would have been assigned “core” if the protein and vegetables consisted of a larger proportion than the fried food.

### Implications for research, policy and practice

While the findings from this study highlight some of the dominant family meal narratives and stereotypes that are perpetuated on social media, investigations are needed into how these representations and messages impact parents’ behaviors and practices. Evidence from this study and the literature suggest that social media may be a useful channel for promoting realistic family meals, healthy eating and evidence informed practices, but more work is required to understand how social media can best be used to promote healthful behaviors. Despite social media’s potential for nutrition and health promotion, there is also the potential for it to cause harm, for example, through propagation of misinformation, promotion of unrealistic standards and its addictive features. Understanding how parents and other individuals use social media to gather information about food and nutrition and how food and nutrition-related content influences psychosocial factors and eating behaviors should be a research priority. Finally, the large proportion of women in the posts examined suggests that preparation of family meals is viewed as “women’s work” and programs and policies to promote men’s cooking skills and encourage a balanced distribution of domestic labor are needed.

## CONCLUSION

This content analysis of Instagram posts about family meals found that meal preparation and mealtimes were predominantly depicted as polished and curated. Posts about family meals also appeared to mainly depict and be authored by women and mothers. These findings suggest that Instagram content reinforces unattainable standards and normative gender roles related to food, which may add to the pressure felt by parents regarding food preparation and family mealtimes. Posts captured in this content analysis also contained meal ideas and recipes and primarily depicted core foods, indicating that Instagram may be a useful resource to support parents in planning healthy meals for their families. Studies that examine how social media content about food influences parents’ eating behaviors, food choice, and psychosocial factors should be a research priority.

## Supplementary Material

daaf078_Supplementary_Data

## Data Availability

The data are not able to be made openly available to the public due to the nature of the data, the conditions of ethics approval, and privacy concerns. Data may be made available via reasonable request to the authors in which a formal data-sharing agreement will be required.
