# Supplementary material for: From plate to post: exploring representations of #familymeals through a content analysis of Instagram
Source: Health Promot Int. 2025 Jun 11;40(3):daaf078. doi: 10.1093/heapro/daaf078 (PMC12154203; doi:10.1093/heapro/daaf078)
Supplement: daaf078_Supplementary_Data [file daaf078_supplementary_data.zip › Supplementary table 1.docx]

**Supplementary Table 1.** Coding framework for Instagram accounts

| **Parent code** | **Subcodes** | **Description & rules** |
| --- | --- | --- |
| **Gender** | | |
| Woman | - Appears as - Self-identifies as | If pronouns are provided, then "self-identifies" should be selected. If pronouns not provided or gender not specifically mentioned then select "appears as", e.g., if person identifies as a mother/father and no pronouns or gender specified then select "appears as". |
| Man |  |  |
| Non-binary |  |  |
| **Parenting** | | |
| Mother | - Appears as - Self-identifies as | If specified on the account (e.g., in the bio) that the person is a mother or father then "self-identifies" should be selected. If parenthood not specifically mentioned but the person appears to be a parent (e.g., lots of photos of them in family environment with young children) then select "appears as". |
| Father |  |  |
| **Descriptive category** | | |
| Recipe developer | - Appears as - Self-identifies as | Profile primarily includes posts containing recipes and/or self-identifies as a recipe developer on their account. |
| Food blog |  | Profile primarily includes pictures of food e.g., from restaurants and/or documenting what they have eaten, and/or self-identifies as a food blog(ger). Also includes accounts that primarily post meal plans. |
| Prenting blogger |  | Profile primarily includes posts about parenting, including discussing their lived experience of parenting. Also includes tips about parenting, feeding kids etc. and/or self-identifies as a parenting blog(ger). |
| Weight loss |  | Profile primarily contains posts about weight loss and/or calorie counting or self-identifies as being about weight loss. |
| General |  | All other categories of accounts, e.g., normal person, lifestyle influencer, travel influencer. |
| Public figure |  | Profile self identifies as being a "public figure" account and/or accountholder has a public facing role e.g., TV presenter, media personality. |
| Dietitian/nutritionist |  | Explicitly identifies as a dietitian or nutritionist on their profile (self-identify only). |
