# Supplementary material for: From plate to post: exploring representations of #familymeals through a content analysis of Instagram
Source: Health Promot Int. 2025 Jun 11;40(3):daaf078. doi: 10.1093/heapro/daaf078 (PMC12154203; doi:10.1093/heapro/daaf078)
Supplement: daaf078_Supplementary_Data [file daaf078_supplementary_data.zip › Supplementary table 2.docx]

**Supplementary Table 2.** Coding framework for Instagram posts

| **Parent code** | **Subcodes** | **Description & rules** |
| --- | --- | --- |
| **Visual elements (images/videos)** | | |
| Food/drink | Cooking | Image/video of someone preparing food. |
|  | Recipe | Instructional images/video of how to prepare a meal (e.g., bows of ingredients, pictures or videos of steps). |
|  | Discretionary food* | Food pictured appears to be predominantly discretionary according to Australian Dietary Guidelines. |
|  | Core food* | Food pictured appears to be predominantly from the five core food groups defined by the Australian Dietary Guidelines (fruit; vegetables; grains; meat and alternatives; milk, cheese and yoghurt). |
|  | Plated up food | Image/video of plated up food (food that has been served on a plate or serving dish). |
|  | Restaurant meal | Meal in image/video from a restaurant or in a restaurant. |
| Sharing a meal | At home* | Image/video pictures people sharing a meal together and appears to be within someone’s home. |
|  | Outside of home* | Image/video pictures people sharing a meal together and appears to be outside the home environment (e.g., at a restaurant, at work, in a park). |
|  | Engaging in celebration | Image/video pictures people sharing a meal together and appears to be a celebration (e.g., people sharing a birthday cake with candles). |
| People depicted | One person* | One person depicted. |
|  | Multiple people* | More than one person depicted. |
|  | Adult | At least one adult is depicted. |
|  | Family | Group of people depicted appear to be a family. |
|  | Child/children | A child or children are depicted. |
|  | Man | Only code gender for adults pictured, not children. Gender assumed to be "appears as" because it is based on what is depicted in the image/video. Gender to be coded based on physical attributes associated with femininity/masculinity. |
|  | Woman |  |
| Tablescape | N/A | Image/video of set table with an emphasis on the setup of the table, plates, etc. |
| Appears staged^ | N/A | Visual aspects related to food/mealtimes appear staged/polished/glamorised/curated (e.g., nicely plated food, perfectly set table with centrepieces etc.). |
| Appears authentic^ | N/A | Visual aspects seem to be authentic to a true cooking/eating experience that has not been dramatically altered to appear a certain way. |
| Not relevant^+^ | N/A | Contents of the video/image not relevant to mealtimes, food or meal preparation. |
| **Caption** | | |
| Meal ideas | Comfort food | Caption contains meal idea (including recipes) described as comfort food. |
|  | Healthy | Caption contains meal idea (including recipes) described as healthy. |
|  | Quick/easy | Caption contains meal idea (including recipes) described as quick/easy. |
|  | Budget friendly | Caption contains meal idea (including recipes) described as budget friendly. |
|  | Kid/family friendly | Caption contains meal idea (including recipes) described as child/kid and/or family friendly. |
|  | Fussy eater friendly | Caption contains meal idea (including recipes) described as fussy eater friendly. |
|  | Recipe in post | Caption contains a recipe. |
|  | Recipe elsewhere | Caption contains a meal idea, and the recipe is hosted elsewhere (e.g., post contains link or directs people to a link in bio for the recipe). |
|  | Freezer friendly | Caption contains meal idea (including recipes) described as freezer friendly. |
|  | “Healthy” alternative | Caption contains meal idea (including recipes) that is described as a healthy alternative to another recipe and/or recipe in caption has ingredients that have been modified to be healthier (e.g., butter replaced by yoghurt in muffins or cake). |
| Food descriptions | Taste | Caption describes the taste of a food. |
|  | Smell | Caption describes the smell of a food. |
|  | Aesthetic | Caption describes the aesthetic/visual appearance of a food. |
|  | Texture | Caption describes the texture/mouthfeel of a food. |
| Food planning | Bulk cooking | Caption contains information about bulk/batch cooking. |
|  | Shopping lists | Caption contains information about writing shopping lists. |
|  | Meal planning | Caption contains information about planning meals. |
| Information/advice | N/A | Caption contains information/advice about mealtimes, food or meal preparation not captured under the “food planning” codes (e.g., healthy eating, strategies for fussy eaters). |
| Celebrations | N/A | Caption describes sharing a family meal for a celebration (e.g., birthday, religious or cultural holiday). |
| Traditions | N/A | Caption describes or discusses some sort of tradition or ritual around meals/cooking (e.g., a specific meal always made for a holiday). Distinct from “celebrations” code which is about sharing meals for celebrations. This code relates to meal preparation. |
| Experience of mealtimes | N/A | Caption describes the experience of mealtimes (e.g., post may describe a child's behaviour during the meal, what they ate or refused to eat, the chaotic nature of mealtimes, difficulties with mealtimes etc.). |
| Emotions/feelings | N/A | Caption contains information about emotions related to mealtimes, food or meal preparation (e.g., excited to share a meal). |
| Not relevant^+^ | Not in English | Caption not written in English. |
|  | Not relevant | Caption content is not relevant to mealtimes, food or meal preparation. |
|  | No caption | Caption does not include any text other than hashtags. |

*Only one of these subcodes could be selected under the relevant parent code.
^Parent codes are mutually exclusive and only one can be selected for a post.
^+^“Not relevant” parent code is exclusive of all other parent codes and if selected this can be the only parent code used.
